# Effect of Various Preterm Infant Milk Formulas on NEC-Like Gut Injury in Mice

**DOI:** 10.3389/fped.2022.902798

**Published:** 2022-07-06

**Authors:** Karishma Rao, Alain Cuna, Susana Chavez-Bueno, Heather Menden, Wei Yu, Ishfaq Ahmed, Pugazhendhi Srinivasan, Shahid Umar, Venkatesh Sampath

**Affiliations:** ^1^Division of Neonatology, Children’s Mercy Hospital, Kansas City, MO, United States; ^2^School of Medicine, University of Missouri-Kansas City, Kansas City, MO, United States; ^3^Division of Infectious Disease, Children’s Mercy Hospital, Kansas City, MO, United States; ^4^Department of Biology, Kansas City Kansas Community College, Kansas City, KS, United States; ^5^Department of Urology, University of Kansas Medical Center, Kansas City, KS, United States; ^6^Department of Surgery, University of Kansas Medical Center, Kansas City, KS, United States

**Keywords:** necrotizing enterocolitis (NEC), probiotics, intestinal microbiome, prematurity, formula

## Abstract

Formula feeding is an important risk factor for the development of necrotizing enterocolitis in preterm infants. The potential harmful effects of different preterm formulas on the developing intestinal tract remain incompletely understood. Here we demonstrate that feeding newborn mouse pups with various preterm formulas resulted in differing effects on intestinal inflammation, apoptosis, and activation of the pro-inflammatory transcription factor NFκB. 16S rRNA sequencing revealed that each preterm formula resulted in significant gut microbial alterations that were different from dam-fed controls. Formula feeding with EleCare and Similac Special Care caused greater intestinal injury compared to NeoSure. Pre-treatment with *Lactobacillus rhamnosus* GG ameliorated severity of intestinal injury from EleCare and Similac Special Care. Our findings indicate that not all preterm formulas are the same, and different formulations can have varying effects on intestinal inflammation, apoptosis, and microbiome composition.

## Introduction

Breastmilk is the nutritional standard for infants, including those born prematurely. The benefits of breastmilk in preterm infants include decreased rates of necrotizing enterocolitis (NEC), late-onset sepsis, and improved neurodevelopmental outcomes (1, 2). Despite these benefits, formula feeding remains common particularly when mother’s own milk or donor milk is unavailable (3, 4). But the use of formula in preterm infants is not benign, as formula feeding is an important risk factor for NEC (5–7).

Several studies have investigated the effects of formula on NEC (8, 9). However, these studies were limited by their use of puppy milk or term milk formula instead of preterm formula. In addition, not all preterm formulas are alike, and each formulation has slightly different characteristics and nutritional content to accomplish its intended goal. For example, Similac Special Care (SSC) is designed to provide the nutritional requirements of convalescing preterm infants admitted to the neonatal intensive care unit (NICU), while Neosure (N) is designed to maintain catch-up growth of preterm infants discharged to home. To promote the higher nutritional requirements of convalescing preterm infants in the NICU, SSC has higher protein, medium chain triglyceride concentration, vitamins, calcium, and phosphorus content than N. On the other hand, EleCare (E) is a hypoallergenic formula often used in preterm infants who do not tolerate standard formulas like SSC or N, typically in the setting of food-protein induced enterocolitis. Thus, the protein in E is broken down to the simplest amino acids whereas the protein in SSC and N is comprised of complex chains of amino acids from non-fat milk and whey protein concentrate. The carbohydrate content of E is also different from SSC or N. E only has corn syrup solids as carbohydrate source whereas SSC and N has both corn syrup solids and lactose. These differences in composition among preterm formulas and their impact on intestinal injury and the developing gut microbiome have not been examined.

Probiotics are live microorganisms that can be beneficial to the host when ingested. Several randomized clinical trials and meta-analyses have provided evidence that probiotic supplementation is safe and well-tolerated in preterm infants (10–12). Moreover, these studies have demonstrated that probiotics can be effective in decreasing the risk for NEC, and may also have benefit in decreasing late-onset sepsis. While its use in clinical practice is starting to increase (13), the “one-size-fits-all” approach of current probiotic intervention remains a major challenge that contributes to inconsistent results (14). As we move more toward individualized medicine, one important question is whether the efficacy of probiotics in protecting against gut injury is similar with different preterm formulas.

In this study, we hypothesized that various preterm formulas have differential effects in causing neonatal gut injury and intestinal microbiome alterations; and that probiotics remain effective in attenuating gut injury caused by different preterm formulas. We investigated this hypothesis by using a newborn mouse model of experimental intestinal injury induced by formula feeding alone with SSC, N, or E. We chose these preterm formulas because SSC and N are standard formulas for preterm infants while in-patient in the NICU and post-discharge, respectively; while E is the first line alternative formula for preterm infants who do not tolerate SSC or N. We chose the probiotic *Lactobacillus rhamnosus GG* (LGG) because it is one of the most studied and widely used probiotics in preterm infants (15–17).

## Methods

### Mice

C57BL/6 mice were obtained from Charles River (Wilmington, MA, United States) and allowed to breed and deliver naturally. All animal experiments were approved by the local Institutional Animal Care and Usage Committee (Protocol # 1601).

### Formula Feeding Experiments

Our protocol for formula-feeding was based on the mouse model of NEC that we and others have used, as previously described (18–20). Briefly, pups from the same litter were randomly assigned to control or formula-feeding groups. Control pups remained with their mothers and breast-fed *ad lib*. Formula-feeding pups were separated from their mothers on postnatal day (P)8, housed in an incubator, and gavage-fed with 0.2 mL of fortified formula (26 kCal/oz) five times daily for 3 days. For an average mouse pup weighing 5 grams, this formula-feeding protocol amounted to about 200 mL/kg/day of fluid volume, 170 kcal/kg/day of calories, and 5 g/kg/day of protein. We tested 3 formula-feeding groups: SSC, N, and E.

### Probiotic Experiments

LGG was obtained from American Type Culture Collection (ATCC 53103, Manassas, VA, United States) and grown per manufacturer recommendations. LGG in de Man, Rogosa and Sharpe (MRS) culture media was harvested by centrifugation, resuspended in PBS/0.1% gelatin, and diluted to 10^8^CFU/ml. We tested 2 probiotic experimental groups: SSC + LGG and E + LGG. Probiotic-treated pups were gavaged with 0.1 mL of 10^8^CFU/ml of LGG once daily from P5 to P7, followed by formula-feeding starting from P8 to P10.

### Sample Collection and Tissue Processing

Pups from both formula-feeding and probiotic experiments were sacrificed on P11 and distal ileum was collected for histology, immunofluorescence, qRT-PCR, and Western blot studies. The distal colon with stool was harvested and immediately frozen for 16s rRNA sequencing under maximal aseptic precautions.

### Histological Grading of Tissue Injury

Histology slides were stained with hematoxylin and eosin and scanned into a computer using a Leica Biosystems Slide Scanner. A standardized 4-point scale (19) was used to grade intestinal injury by two blinded investigators (KR and WY).

### TUNEL Assay

Terminal deoxynucleotidyl transferase dUTP nick end labeling (TUNEL) assay was performed on terminal ileum as previously described (21). The apoptotic index was calculated by dividing the total number of TUNEL positive cells by the total number of DAPI stained cells. A minimum of 2 slides per mouse and 3–5 fields per slide were analyzed.

### qRT-PCR

Gene expression data were collected on a Bio-Rad iQ5 with SYBR Green Mastermix using pre-validated primers from MilliporeSigma (St. Louis, MO, United States). Relative gene expression of interleukin 1 beta (IL1B), toll like receptor 4 (TLR4), single immunoglobulin and interleukin-1-related receptor (SIGIRR), and inducible nitric oxide synthase (iNOS) was calculated with the Pfaffl method (22), with housekeeping gene 18S rRNA for normalization.

### Western Blot

Ileal samples were lysed in radioimmunoprecipitation assay (RIPA) buffer and then homogenized using a bullet blender. Immunoblotting was done following standard protocols as previously described (21). The antibodies used were as follows: rabbit anti-(p) p38 mitogen-activated protein kinases (MAPK), rabbit anti-p38 MAPK, rabbit anti-(p) p65, mouse anti-ICAM-1, rabbit anti-Cleaved Caspase 3 (CC3), rabbit anti-p65, and mouse anti-ß-Actin. ß-actin was used as a loading control. Blots were detected by chemiluminescence and densitometry was quantified using ImageJ software.

### 16s rRNA Sequencing

Total bacterial genomic DNA was extracted using the QIAmp DNA stool kit (Qiagen) following their instructions. The DNA integrity was assessed by agarose gel electrophoresis; concentration and quality were determined by absorption at A260, and A260/A280 ratio, respectively, using a Nanodrop-2000 spectrophotometer (Thermo Fisher Scientific). The 16S V4 region was amplified using 515F/806R primers and sequenced using amplicon sequencing on IonS5™XL to generate raw reads. Paired-end reads were assigned to samples based on their unique barcode and truncated by cutting off the barcode and primer sequences. We used Cutadapt (23) (V2.1^1^) with parameters p-error-rate 0.1 to remove primers and adaptors from the sequences before performing downstream bioinformatic processes in QIIME2_v2020.6. Briefly, we used QIIME2-wrapped DADA2 (24) (v1.14) to remove chimeric and singleton sequences and join paired-end reads to provide the amplicon sequence variants (ASV) table. Resulting sequences were assigned taxonomy using a pre-trained Naïve Bayes classifier trained on the Greengenes 13_8_ 99% OTUs. Phylogenetic tree was generated using align-to-tree-mafft-fasttree pipeline from the q2-phylogeny plugin.

### Data Analysis

Analysis of variance with Bonferroni correction for multiple testing was used for analysis. Statistical analysis was done using GraphPad Prism (San Diego, CA, United States) with statistical significance set at *P* < 0.05. For microbiome analysis, microbial community structures were characterized using measures of α- (within-sample) and β-(between-samples) diversity indices. α-diversity was applied in analyzing the complexity of species diversity for a sample through indices including observed richness, Shannon, ACE and InvSimpson. B eta-diversity (Bray-Curtis dissimilarity index) assessed between-sample and between-group species diversity that was visualized with a Principal Coordinate Analysis (PCoA) plot. Linear discriminant analysis effect size (LEfSe) was used to investigate the differences in bacterial and pathway abundances between control and formula groups (25). LEfSe incorporates the Kruskal–Wallis rank-sum test to identify features with significant differences between groups and subsequent linear discriminant analysis to estimate the effect size of the feature of interest.

## Results

### Formula-Feeding With Similac Special Care and EleCare Caused Greater Injury Than NeoSure

**FIGURE 1:**
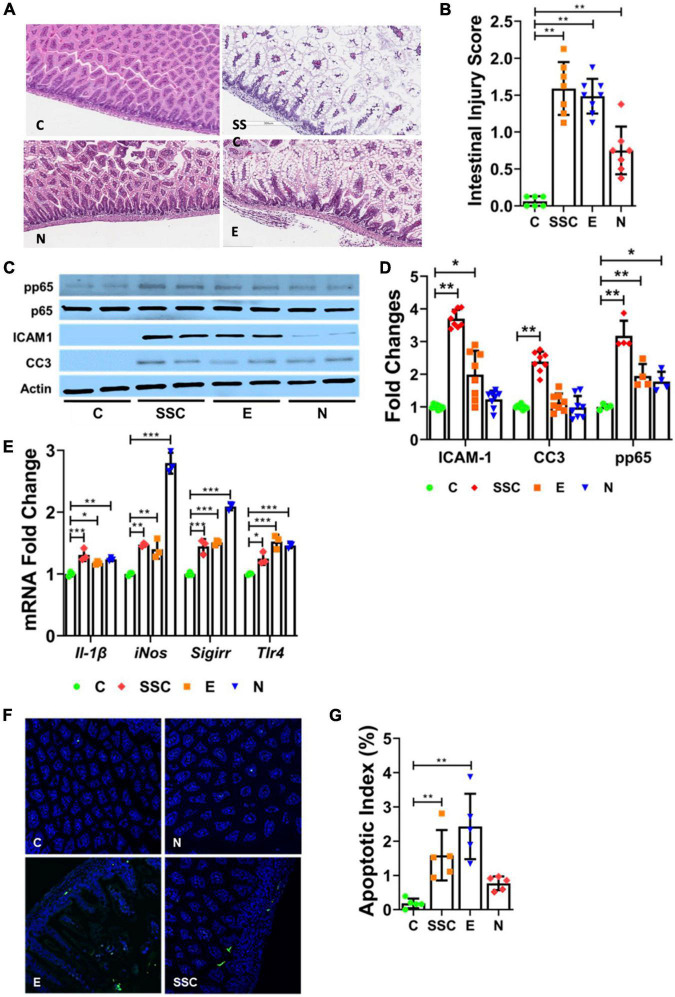
Preterm formula elicits injury to the neonatal intestinal tract. **(A)** Representative histologic images of the terminal ileum from control (C) and formula-fed mouse pups (SSC, Similac Special Care; N, Neosure; E, EleCare). **(B)** Histologic intestinal injury scores. **(C,D)** Western Blot showing protein expression of phosphorylated p65, ICAM1, and CC3 from ileal tissue of control and formula-fed mouse pups. **(E)** Relative gene expression of IL-1β, iNOS, SIGIRR, and TLR4 in controls vs. formula-fed pups. **(F)** TUNEL staining of terminal ileum of control and formula-fed groups. Green indicates TUNEL positive cells; blue, DAPI cells. **(G)** Apoptotic index indicating ratio of TUNEL positive cells to DAPI cells. All data are presented as mean ± SD; *P < 0.05, **P < 0.01, ***P < 0.001 by ANOVA. *n* = 7–11 pups in each group.

We evaluated ileal histology of control and formula-fed mouse pups to determine the effects of different preterm formulas on the intestinal tract. We found that feeding with preterm formula alone was sufficient to cause mild to moderate injury to the intestinal tract (Figure 1A). In comparing the extent of injury from different preterm formulas, we observed that SSC and E were similar in causing significant intestinal injury compared to controls, while N resulted in modest intestinal injury (Figure 1B).

We next investigated whether differences in gut injury were associated with changes in pro-inflammatory TLR-mediated NFκB activation, a known key mediator of inflammation in NEC (13, 14). We found that formula-feeding resulted in higher protein expression of intercellular adhesion molecule 1 (ICAM1) and phosphorylated p65 (p65, also known as RelA, a subunit of NFκB transcription factor complex) compared to dam-fed controls (Figure 1C). The level of protein expression of ICAM1 and phosphorylated p65 correlated with the level of intestinal injury from formula feeding (Figure 1D). We also investigated gene expression of pro-inflammatory genes from terminal ileal lysates and found modest induction of IL1B and TLR4 in formula-fed groups compared to controls (Figure 1E).

We then evaluated gene expression of gut protective genes SIGIRR and iNOS. SIGIRR is a TLR antagonist that protects against exaggerated TLR activation in NEC (21), while iNOS is a key mediator of early villous re-epithelialization following acute mucosal injury (26). Interestingly, we found significant induction of both SIGIRR and iNOS among pups fed with NeoSure, which correlated with the modest gut injury found in this group (Figure 1E).

Lastly, we determined the effects of different preterm formulas on intestinal apoptosis by CC3 protein expression (Figures 1C,D) and TUNEL assay (Figures 1F,G). We found a similar pattern of increased apoptosis among pups in the SSC and E groups, while pups in the N group demonstrated comparable CC3 protein expression and apoptotic index as control pups (Figure 1G). Taken together, our findings indicate differing effects of preterm formulas on intestinal injury, inflammation, and apoptosis—with Similac Special Care and EleCare causing greater intestinal injury than NeoSure.

### Each Preterm Formula Caused Distinct Alterations to the Developing Gut Microbiome

**FIGURE 2:**
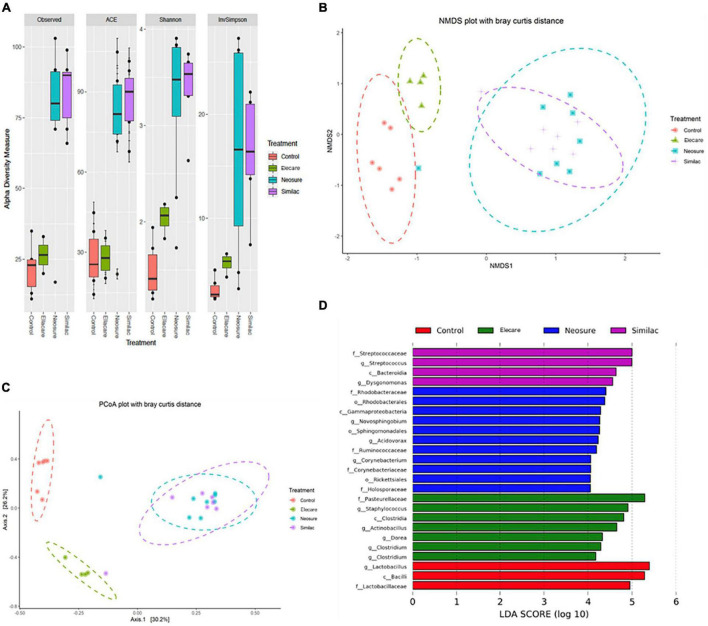
Preterm formula influences the acquisition of the intestinal microbiome. **(A)** Alpha diversity calculations with Phyloseq software package. Standard diversity metrics were evaluated including observed richness, ACE, Shannon index, and InvSimpson. **(B,C)** Beta-diversity visualization with Non-metric Multi-dimensional Scaling (NMDS) and Principal Coordinate Analysis (PCoA) plots based on Bray-Curtis distances. **(D)** The linear discriminant analysis (LDA) value distribution histogram. Taxa meeting a linear discriminant analysis significant threshold > 4 are shown. c: class level; f: family level; g: genus level; o: order level; p: phylum level.

To further explore additional mechanisms related to intestinal injury elicited by different preterm formulas, we investigated whether differences in microbial composition could underlie differences in NEC-like injury. We collected colonic stool at the time of pup sacrifice and evaluated alterations in microbial community composition and diversity (Figure 2A). We investigated α-diversity within the groups using Phyloseq (27). Standard α-diversity metrics were evaluated, including observed richness, ACE, Shannon index and InvSimpson. To visualize the results for β-diversity, Non-metric Multi-dimensional (NMDS) plots (Figure 2B) and PCoA plots (Figure 2C) were generated based on Bray-Curtis distances. Bray-Curtis provides a measure of community composition differences between samples based on OTU counts, regardless of taxonomic assignment.

Our results showed that N (*n* = 8) and SSC (*n* = 7) groups exhibited significantly different α-diversity measures (*P* < 0.01) compared to dam-fed controls, while pups in E group (*n* = 8) exhibited only modest differences (*P* < 0.05) (Figure 2A). The ordinations based on β-diversity metrics followed a similar pattern, with clear clustering of N and SSC samples that is distinctly different from controls (*P* < 0.001), while separation between control and E samples (Figures 2B,C) was less obvious. Interestingly, we found poor correlation between α- and β-diversity measures and severity of gut injury. Pups in E group demonstrated significant gut injury despite the close similarity in α- and β-diversity measures with dam-fed controls. Contrasting effects on gut injury in N and SSC group (less injury with N, more injury with SSC) were also noted despite the similarities in α- and β-diversity measures between the two groups.

We also conducted LEfSe analysis to identify species with significant differences among the formula groups (Figure 2D). LEfSe statistical results include three parts, namely, the linear discriminant analysis (LDA) value distribution histogram, evolutionary branch graph (phylogenetic distribution), and biomarker abundance comparison chart in different groups. The LEfSe was used to identify discriminative bacterial taxon among different groups. LDA (log10 > 4) and LEfSe analysis revealed significant differences (*P* < 0.05) in the fecal microbiota exhibited by indicated groups (Figure 2D). Several bacterial species commonly regarded as potentially pathogenic were identified among formula-fed groups including *Streptococcaceae*, *Gammaproteobacteria*, *Corynebacterium*, *Staphylococcus*, *Clostridia*, and *Actinobacillus*. In contrast, the commensal bacterial species *Lactobacillus*, *Bacilli*, and *Lactobacillaceae* were found to predominate in dam-fed controls.

### LGG Pre-treatment Ameliorated Formula-Feeding Injury Caused by Similac Special Care and EleCare

**FIGURE 3:**
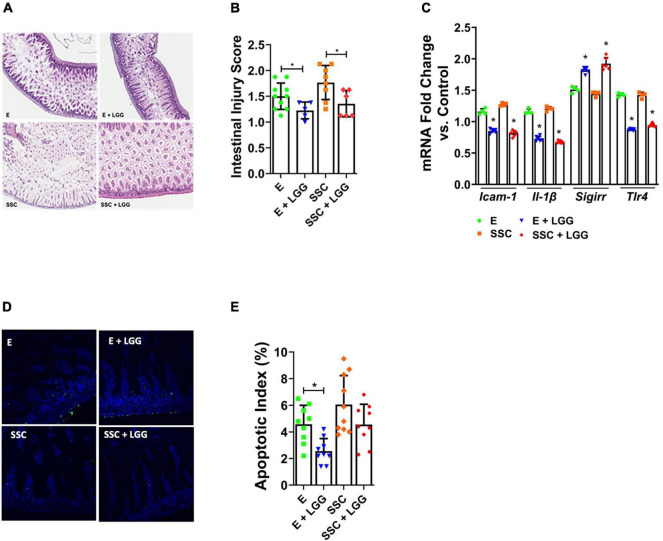
Lactobacillus rhamnosus GG (LGG) ameliorates intestinal injury by preterm formulas. **(A)** Representative histologic slides of the terminal ileum of formula-fed pups with and without pre-treatment with LGG. **(B)** Histologic intestinal injury scores by blinded investigators. **(C)** Relative gene expression of ICAM1, IL-1β, SIGIRR, and TLR4. **(D)** TUNEL staining of terminal ileum comparing formula versus formula + LGG. **(E)** Quantification of apoptosis by calculating apoptotic index (ratio of TUNEL positive cells to DAPI cells). Data presented as mean ± SD; *P < 0.05, by *t*-test. *n* = 6 pups in each group.

We next investigated whether LGG—a probiotic that protects against experimental and human NEC—will be protective against intestinal injury from preterm formula. We focused our probiotic experiments on EleCare and Similac Special Care since NeoSure-fed mice exhibited only low levels of injury (28). We found that pre-treatment with LGG significantly reduced histologic gut injury (Figures 3A,B) and decreased gene expression of pro-inflammatory mediators ICAM-1, IL-1B, and TLR4 (Figure 3C) in E and SSC groups. We also observed that LGG significantly induced SIGIRR gene expression (Figure 3C). This observation, which we also demonstrated in our previous study (18), indicate that SIGIRR induction may be one of the mechanisms by which LGG protects against intestinal injury from preterm formula. Lastly, we found that LGG was also effective in decreasing apoptotic injury caused by E (Figures 3D,E). Taken together, these findings indicate that LGG remains effective in ameliorating formula-induced injury despite the varying effects of SSC and E on the gut microbiome.

## Discussion

In this study we have shown that different preterm formulas cause varying injury to the neonatal mouse gut. More specifically, we found that SSC (a specialized high-protein preterm formula) and E (an elemental infant formula) caused greater gut injury, apoptosis, and pro-inflammatory NFκB activation than N (a preterm post-discharge formula). We also found that the different preterm formulas caused distinct alterations in gut microbial composition, and that pre-treatment with LGG was effective at ameliorating the level of injury from formula-feeding. Taken together, these data highlight that not all preterm formulas are the same, and that subtle differences in their composition can have varying effects on neonatal gut injury and the developing gut microbiome.

Differences in nutrient content among the formulas we tested could explain some of the differences in gut injury and gut microbiome we observed in the study. For example, the carbohydrate content in SSC and N include lactose, whereas E does not. Lactose is a preferred carbon source of *Bifidobacterium* and helps promote the growth of this commensal bacteria in the gut (29). The absence of lactose in E can thus decrease growth of *Bifidobacterium* and increase susceptibility of E-fed pups toward injury in the gut. In addition to carbohydrates, gut bacteria also metabolize dietary proteins and produce metabolites that are mutually beneficial to bacterial communities and the host (30–32). The completely hydrolyzed protein content in E can thus have a different impact on the gut microbiome compared to the intact protein content in SSC or N. This theory was tested by Kok et al. (33). in a randomized, double-blinded trial comparing stool microbiota of term infants fed with intact, extensively hydrolyzed, or completely hydrolyzed milk. They found that infants receiving extensively hydrolyzed or completely hydrolyzed milk developed patterns of early gut microbiome that was distinctly different from infants fed with intact milk. Interestingly, among the patterns they observed was an increased abundance of *Clostridia* with hydrolyzed protein formula. Increased *Clostridia*, which we also found in pups who received E, has been identified in microbiome studies of preterm infants to be associated with cases of NEC (34). Overall, our findings suggest that differences in nutrient content of formulas can impact the balance between commensal and pathogenic gut bacterial communities and influence susceptibility of the developing neonatal gut to injury.

A number of microbial taxa we identified as differentially enriched in dam-fed versus formula-fed groups were consistent with reports of health and NEC in infants. For example, *Lactobacillus*, differentially increased in healthy dam-fed pups, is a commensal bacteria often used as a probiotic to reduce NEC in preterm infants (17). Conversely, *Pasteurella* and *Clostridia*, differentially increased in E-fed mice that had severe gut injury, are pathogenic bacteria identified in microbiome studies to be associated with NEC in preterm infants (34, 35). Yet not all the changes in gut microbial taxa we observed were consistent with the expected patterns of injury based on existing literature. For example, increased *Gammaproteobacteria*, observed in mice that received N, has been identified in several studies to precede NEC development in humans (36, 37). Yet in our study, N caused the least amount of injury compared to SSC or E. Similarly, the α- and β-diversity measures of E-fed pups were similar with dam-fed controls. Yet despite this similarity with healthy controls, pups who received E exhibited severe gut injury. These inconsistent findings between gut microbiota changes and gut injury suggest that other mechanisms can regulate injury caused by formula feeding besides the gut microbiome. One possible mechanism is that of increased osmolality causing direct injury to the intestinal mucosa. In comparing the different formulas, the osmolality of E (455 mOsm/kg) is significantly higher compared to N (295 mOsm/kg) and SSC (303 mOsm/kg), and is above the 450 mOsm/kg threshold recommended by the American Academy of Pediatrics for enteral nutrition (38). This higher osmolality may partly explain the intestinal injury elicited by E, despite the similarities in α- and β-diversity of E-fed pups with dam-fed controls (39–41). Similar findings of gut injury dependent on osmolality and not on microbial changes were also demonstrated by the study of Lueschow et al. (42). Another possible mechanism is that of increased induction of beneficial genes such as SIGIRR and iNOS protecting against gut injury. SIGIRR inhibits intestinal TLR activity and can protect against excessive TLR-mediated intestinal inflammation (21), while iNOS mediates early intestinal epithelial repair following acute mucosal injury (26). The increased induction of SIGIRR and iNOS by N could thus explain the lower injury scores observed in N-fed pups despite their increased *Gammaproteobacteria*. Taken together, these results highlight the complexity of the gut microbiome and how associative changes in gut microbial diversity and composition do not necessarily correlate with phenotype. Additional studies combining metagenomics with other multi-omics approaches are needed to further investigate the impact of formula-driven differences in gut microbiome on health and disease.

The study by Repa et al. (43). suggested that probiotics may be protective against NEC in breast-fed but not formula-fed infants. Contrary to their findings, our current study provides evidence that the probiotic LGG remained effective in decreasing gut injury from various preterm formulas. The mechanisms by which probiotics protect against gut injury remain poorly understood. However, recent studies indicate that overt changes in the gut microbiome may not be the main mechanism by which probiotics confer its beneficial effects (44, 45). Using both mice and humans, Zmora et al. demonstrated that probiotic treatment caused only minimal changes in gut bacterial composition compared to placebo-treatment or to their own pre-probiotic baseline (44). Instead, what they found was that probiotic supplementation caused marked differences in mRNA expression of mostly immune-related genes in the intestinal mucosa. Based on their findings, the authors concluded that probiotics confer benefits by regulating gene expression rather than by modulating the composition of gut bacterial communities. Consistent with the study by Zmora et al., our previous study demonstrated that the probiotic LGG conferred protection in mice subjected to formula-feeding and experimental NEC injury despite not having major changes in gut microbiome composition (18). Instead, in both this previous study and the current study, we found that LGG administration induced mRNA expression of the gut protective gene SIGIRR. Taken together, these results provide evidence that probiotics can reduce intestinal injury even when artificial enteral nutrition is administered. Moreover, the mechanism by which probiotics confer this protection maybe mediated by inducing gut protective genes such as SIGIRR rather than by modulating the composition of gut bacterial communities.

Murine models are often used to study NEC (46), but their use of Esbilac (a milk replacement for puppies and dogs) or Similac Advance (a term infant formula) to elicit gut injury may have important limitations (19, 20, 47). For example, current formulations of Esbilac contain probiotics and arginine—dietary supplements that have been shown in studies to prevent NEC (11, 48–50). The presence of these dietary supplements may thus decrease the effectiveness of Esbilac in inducing NEC. Similarly, term formula contains less nutrients, vitamins, and minerals—and consequently decreased osmolarity—compared to preterm formula. As administration of hyperosmolar formula is regarded as an important risk factor for NEC, term formula may also be less effective in inducing NEC compared to preterm formula. In our study, we investigated intestinal injury arising from different preterm formulas commonly used in the NICU. In addition, instead of the traditional formula plus hypoxia plus enteric endotoxin model, we used a formula-fed only model to limit the effect of confounding from hypoxia and enteral endotoxin or bacteria. Even with this less aggressive model, we found that preterm formula was sufficient to elicit gut injury in neonatal mice in the absence of hypoxia or enteral endotoxin administration. Moreover, the observed changes in gut injury were associated with increased cell death and increased pro-inflammatory NFκB activity, key characteristics that mimic injury seen in human and experimental NEC (20). Taken together, our results suggest that using preterm formula instead of Esbilac or term formula can be an appropriate alternative to current experimental NEC models.

While our study compared three commonly used preterm formulas, we acknowledge that there are several other preterm milk formulations that we did not test. Our study was also limited to the effects of preterm formula in newborn mice. It would require large studies to determine whether different preterm formulas have varying abilities to cause intestinal injury in preterm infants.

Formula-feeding in preterm infants is generally accepted to be less beneficial compared to breastmilk. However, several different types of formula are used in preterm infants; each with its own unique blend of nutrients, minerals, and additives. Our study addresses a significant gap in knowledge regarding the differential injury caused by different preterm formulas. Further studies are needed to elucidate the effects of different preterm formulas on the intestinal tract of infants.

## Data Availability Statement

The original contributions presented in this study are publicly available. This data can be found here: BioProject: PRJNA838402.

## Ethics Statement

The animal study was reviewed and approved by Institutional Animal Care and Usage Committee at University of Missouri-Kansas City.

## Author Contributions

KR helped conceptualize and plan the experiments, performed the experiments, helped with the analysis, co-wrote the first draft of the manuscript, and contributed to the final version of the manuscript. AC helped conceptualize and plan the experiments, helped with the analysis, co-wrote the first draft of the manuscript, and contributed to the final version of the manuscript. HM and WY performed the experiments, helped with the analysis and figures, and contributed to the final version of the manuscript. SC-B performed the probiotics culture and quantification, helped with the figures, and contributed to the final version of the manuscript. IA, PS, and SU performed the 16S microbiome sequencing and analysis, helped with the figures, and contributed to the final version of the manuscript. VS conceived the original idea, helped conceptualize and plan the experiments, helped with analysis and interpretation of results, supervised the project, and contributed to the final manuscript. All authors contributed to the article and approved the submitted version.

## Conflict of Interest

The authors declare that the research was conducted in the absence of any commercial or financial relationships that could be construed as a potential conflict of interest.

## Publisher’s Note

All claims expressed in this article are solely those of the authors and do not necessarily represent those of their affiliated organizations, or those of the publisher, the editors and the reviewers. Any product that may be evaluated in this article, or claim that may be made by its manufacturer, is not guaranteed or endorsed by the publisher.

## Footnotes

1
http://cutadapt.readthedocs.io/en/stable/
